# Nmr-VSM: Non-Touch Motion-Robust Vital Sign Monitoring via UWB Radar Based on Deep Learning

**DOI:** 10.3390/mi14071479

**Published:** 2023-07-24

**Authors:** Zhonghang Yuan, Shuaibing Lu, Yi He, Xuetao Liu, Juan Fang

**Affiliations:** 1 Faculty of Information Technology, Beijing University of Technology, Beijing 100124, China; yuanzh@emails.bjut.edu.cn (Z.Y.); lushuaibing@bjut.edu.cn (S.L.); liuxuetao3616@163.com (X.L.); 2 School of Software Engineering, Beijing Jiaotong University, Beijing 100091, China; 20301038@bjtu.edu.cn

**Keywords:** non-touch vital sign monitoring, ultra-wideband (UWB) radar, multi-dimensional vital sign, heart rate data correction, anomaly detection

## Abstract

In recent years, biometric radar has gained increasing attention in the field of non-touch vital sign monitoring due to its high accuracy and strong ability to detect fine-grained movements. However, most current research on biometric radar can only achieve heart rate or respiration rate monitoring in static environments, which have strict monitoring requirements and single monitoring parameters. Moreover, most studies have not applied the collected data despite their significant potential for applications. In this paper, we introduce a non-touch motion-robust vital sign monitoring system via ultra-wideband (UWB) radar based on deep learning. Nmr-VSM not only enables multi-dimensional vital sign monitoring under human motion environments but also implements cardiac anomaly detection. The design of Nmr-VSM includes three key components. Firstly, we design a UWB radar that can perform multi-dimensional vital sign monitoring, including heart rate, respiratory rate, distance, and motion status. Secondly, we collect real experimental data and analyze the impact of eight factors, such as motion status and distance, on heart rate monitoring. We then propose a deep neural network (DNN)-based heart rate data correction model that achieves high robustness in motion environments. Finally, we model the heart rate variability (HRV) of the human body and propose a convolutional neural network (CNN)-based anomaly detection model that achieves low-latency detection of heart diseases, such as ventricular tachycardia and ventricular fibrillation. Experimental results in a real environment demonstrate that Nmr-VSM can not only accurately monitor heart rate but also achieve anomaly detection with low latency.

## 1. Introduction

Vital sign monitoring system refers to a system that can detect important indicators of physiological health, such as heart rate, respiratory rate, blood pressure, body temperature, and more. These vital signs are crucial indicators of the body’s physiological state and are of significant importance in disease prevention, diagnosis, and treatment.

A vital sign monitoring system can broadly be divided into two categories: touch vital sign monitoring system and non-touch vital sign monitoring system. Touch vital sign monitoring system refers to a system that requires sensors to come in contact with the human body to detect vital signs, such as electrocardiogram monitors, smart bracelets, and heart rate monitors. On the other hand, non-touch vital sign monitoring system refers to a system that can detect vital signs without direct sensor contact with the human body, such as UWB radar and infrared thermometers.

### 1.1. Motivation and Challenges

A vital sign monitoring system is primarily utilized to monitor crucial physiological indicators reflecting the body’s condition, such as heart rate and respiratory rate. By monitoring these physiological indicators, we can obtain insight into a person’s physiological condition, which can serve as a basis for disease diagnosis, ultimately achieving the goal of improving quality of life and health levels. Currently, touch system is the most common option, which offers strong real-time performance and high accuracy; however, its application is limited due to its need for sensors to come into contact with the body. First of all, the use of a touch system might result in a negative subject experience and possibly atypical physiological or psychological reactions, which can contribute to measurement mistakes. Second, employing a touch system can be extremely uncomfortable for the subject in some unique circumstances, such as monitoring vital signs in patients with severe burn injuries, and the detection accuracy may suffer as a result of the subject’s lack of skin tissue [1]. As a result, a non-touch system has emerged as an alternative option.

From a technical point of view, a non-touch system can be based on technologies such as RFID [2], capacitively coupled electrocardiogram [3], speech signals, video imaging, thermal imaging, and biometric radar [4]. Among these, a biometric-radar-based non-touch system has been a recent research focus due to its relatively higher monitoring accuracy and lower costs.

A non-touch vital sign monitoring system based on biometric radar has addressed the pain points of a touch system and has been applied to a certain extent in real life [5]. However, there still exist a number of issues with biometric radar. First, little study has been completed on variables like human motion and distance in current biometric radar research, which mostly focuses on heart rate and respiration rate detection. Second, biometric radar relies on radio waves for monitoring, which makes it susceptible to interference and having poor stability. Therefore, most current research on biometric radar requires the subject to remain motionless. Third, the majority of biometric radars can only monitor vital signs without applying the collected data. These vital sign data actually have a wide range of applications.

In order to address the issues with a non-touch system based on the biometric radar mentioned above, we propose a non-touch motion-robust vital sign monitoring system, Nmr-VSM, which leverages UWB radar to detect multi-dimensional vital sign data, such as heart rate, breathing rate, and human motion. Moreover, we take heart rate monitoring as an example, analyze the real experimental data, and subsequently propose a novel data correction model based on DNN to correct the heart rate data measured by the radar in a motion environment. Finally, we model the heart rate variability (HRV) and propose a CNN-based cardiac anomaly detection model to detect heart diseases.

### 1.2. Contributions and Paper Organization

We introduce a non-touch motion-robust vital sign monitoring system via UWB radar based on deep learning, Nmr-VSM, which addresses common issues currently existing in biometric radar systems, such as limited monitoring parameters, susceptibility to interference, and poor robustness.

We highlight our main contributions as follows:

- We design a UWB radar that can not only monitor human heart rate and respiratory rate in real time but also capture parameters such as human motion status and distance in real time, achieving the monitoring of multi-dimensional vital signs.
- We take the radar’s heart rate monitoring as an example, collect data through real experiments, and subsequently analyze the impact of human motion status on the accuracy of heart rate monitoring. At the same time, we combine variables such as angle, distance, direction, and the subject’s gender, weight, height, etc., and propose a DNN-based heart rate data correction model, which improves the robustness of the system in motion environments.
- We model the HRV of the human body and propose a CNN-based cardiac anomaly detection model. Additionally, we propose a metric, the latency value k, to evaluate the performance of this model. Verification results demonstrate that this model achieves low-latency detection of heart diseases such as ventricular tachycardia and ventricular fibrillation.
- We conduct experiments in a real environment and the results show that, after correcting the heart rate detected by UWB radar, mean absolute error (MAE), mean squared error (MSE), and mean relative error (MRE), compared with before correction, all at least decreased by more than 85%, and the heart rate change trend after correction is more consistent with the real heart rate change trend. In addition, our designed anomaly detection model achieves a high detection success rate with low latency.

The remainder of this paper is organized as follows. Section 2 surveys related works. Section 3 sequentially describes the UWB radar, the data correction model, and the anomaly detection model. Section 4 presents the experiments. Finally, Section 5 concludes the paper.

## 2. Related Work

In this section, we introduce the UWB radar technology, and then summarize the key research in the field of vital sign monitoring.

### 2.1. UWB Radar

UWB radar has been used in fields such as robot navigation [6], indoor positioning [7], map construction [8], etc. The latest research also shows that UWB radar can be applied to autonomous driving [9,10] and human identification [11,12]. All these studies demonstrate the feasibility of applying UWB radar to the field of vital sign monitoring. Along this direction, this paper will leverage UWB radar to achieve non-touch vital sign monitoring.

### 2.2. Vital Sign Monitoring

Some studies use touch technologies for vital sign monitoring, such as [13] based on PPG (photoplethysmography) technology, using smart bracelets to monitor human heart rate and motion, and designed a sleep staging model, but this touch monitoring method may provide users an uncomfortable experience, resulting in abnormal monitoring results. Some other studies [14] based on ECG (electrocardiogram) technology achieved medical-grade electrocardiogram monitoring. However, ECG must use electrodes to touch the human body, so this technology will also provide users an uncomfortable experience. Not only that, the conductive gel used by ECG electrodes may cause allergic reactions in users, and non-medical ECG electrodes are prone to problems, such as displacement or detachment. Most importantly, ECG technology is not suitable for specific populations, such as infants and large-area burn patients.

In order to solve the problems of a touch system and improve user experience, some studies have started to use speech signals, video imaging, WiFi and biometric radar, and other technologies to achieve non-touch vital sign monitoring.

The research of Mesleh A and Skopin D. et al. [15] pointed out that the human heart activity is dynamically correlated with changes in vocal cord parameters, so it is feasible to detect heart rate by extracting appropriate frequency features from human voice signals. They showed the study of vowel voice signals in [15], and the experimental results showed that the average error rate of heart rate detection in this study was 5%. However, when the subject’s vital capacity is insufficient, the detection result of this technique is not satisfactory because the subject’s tone change in the process of speaking is usually very large when the vital capacity is insufficient.

Poh M. Z. et al. [16,17] used a laptop’s camera to achieve non-touch vital sign detection based on video detection. The principle of this technology is that, when ambient light shines on the surface of human skin, the blood volume in the skin will show pulsatile changes with the heartbeat, resulting in skin color changes. By analyzing these color changes, the heart rate can be obtained. However, this technology is very limited by ambient light and can only perform better detection under suitable lighting conditions, and this technology is also very susceptible to motion artefact.

F. Zhang et al. [18] and C. Wu et al. [19] successfully utilized WiFi to achieve human respiratory rate detection; the principle of this technology is detecting chest rise and fall caused by breathing through WiFi signals. However, the current WiFi system’s carrier frequency is usually 2.4 GHz or 5 GHz, which results in a relatively low spatial resolution. Moreover, the narrow bandwidth characteristic of a WiFi system makes its distance resolution low. However, the largest challenge is that, due to the extremely low signal-to-noise ratio of the heartbeat signal, it is difficult for a WiFi system to accurately detect human heartbeats [20].

The basic principle of utilizing biological radar to detect vital signs is similar to WiFi. However, compared to WiFi, radar can have higher carrier frequency and bandwidth, and also a stronger ability to detect weak motion. Therefore, biological radar can not only detect human respiration more accurately but also the human heartbeat. For example, D. Obeid et al. [21] utilized millimeter waves to achieve non-touch heart rate monitoring and heart rate variability extraction, and Y. Lee et al. [22] utilized UWB radar to achieve the same function. Despite the promising results achieved by both studies, due to the physical principles involved in measuring heart rate using biological radar, the subjects in these studies were required to remain still during measurements. Any motion would result in motion artifacts [23] that significantly affect measurement accuracy. To address this problem, the study [24] improved the sensitivity of motion artefacts to some extent by using multiple antennas.

Different from the above studies, this paper designs a non-touch motion-robust vital sign monitoring system via UWB radar based on deep learning, Nmr-VSM, which supports heart rate data correction in motion environments without using multiple antennas, reducing the cost. Moreover, we also design an anomaly detection model to better perform health monitoring.

## 3. System Design

### 3.1. Preliminary

**Physiological background.** Human respiration is accomplished by the periodic expansion and contraction of the diaphragm and chest muscles; similarly, human heartbeat is accomplished by the periodic expansion and contraction of the heart. Therefore, both human respiration and heartbeat activity cause a certain degree of chest rise and fall. Generally speaking, the frequency and amplitude of this chest rise and fall are as shown in Table 1.

**Electromagnetic background.** UWB radar emits radar signals towards the subject, which are reflected by the surface of the chest, thereby forming an echo signal. At this point, the chest rise and fall caused by breathing and heartbeat will affect the phase of the echo signal; this phenomenon is called phase modulation. Assuming that the amplitude of the chest rise and fall of the subject is x(Δt), phase modulation θ(Δt) can be expressed as:

(1) $$\theta \left(\Delta t\right)=\frac{4\pi x(\Delta t)}{\lambda }$$

where Δt represents the time from emitting the radar signal to receiving the echo signal, in seconds. λ represents the wavelength of the emitted radar signal, in meters.

According to Equation (1), the amplitude x(Δt) of the human chest rise and fall is proportional to the phase modulation θ(Δt) of the signal. Therefore, theoretically, as long as an appropriate signal processing method is used to obtain θ(Δt), x(Δt) can be obtained, and then information such as the subject’s heart rate and respiration rate can be obtained from it.

### 3.2. System Overview

**Figure 1:**
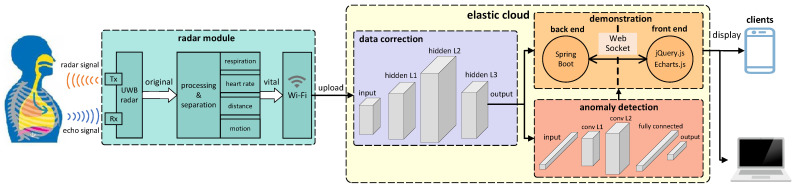
An overview of the Nmr-VSM’s architecture.

We designed a non-touch vital sign monitoring system, Nmr-VSM, which leverages UWB radar to achieve multi-dimensional vital sign monitoring, such as heart rate and respiration rate. The system architecture is shown in Figure 1. The whole system consists of a radar module, data correction, anomaly detection, and demonstration.

### 3.3. Radar Module

Nmr-VSM first preprocesses the echo signal in a computing chip that is in the radar module to detect heart rate, respiratory rate, motion status, and distance. Then, these vital sign data are uploaded to an ECS (Elastic Cloud Server) via WiFi.

**UWB radar.** UWB is a wireless carrier communication technology that utilizes narrow-pulse non-sinusoidal waves with sub-nanosecond durations to transmit data. Operating within the frequency range of 6.5–8.1 GHz, UWB has a transmission bandwidth of over 500 MHz. Additionally, UWB radar has an extremely short transmission duration while the transmitter-receiver cycle time is relatively long, resulting in extremely low power consumption per unit time, typically below 5 dBm.

Nmr-VSM leverages UWB pulse radar to detect the chest rise and fall caused by respiration and heartbeat, which consists of a radio frequency antenna, a radar chip, and a microcontroller unit. Specifically, the radar emits electromagnetic pulse signal towards the human body. Then, the radar uses the receiving antenna to receive the signal whose frequency and phase have been changed, which is the echo signal carrying vital sign information.

**Echo signal preprocessing.** In order to extract vital sign information from the echo signals, a computing chip in the radar module will preprocess the signals to obtain vital sign data. Specifically, due to the periodic expansion and contraction of the chest caused by heartbeat and breathing, the distance d(t) between the chest surface and the radar varies periodically around a baseline distance $d_{0}$. This periodic variation of d(t) can be modeled as:

(2) $$d\left(t\right)=d_{0}+m_{b}\sin\left(2\pi f_{b}t\right)+m_{h}\sin\left(2\pi f_{h}t\right)$$

where $m_{b}$ and $m_{h}$ are the displacement amplitudes of respiration and heartbeat, respectively, and $f_{b}$ and $f_{h}$ are the frequencies of respiration and heartbeat, respectively.

Based on Equation (2), further processing such as denoising and filtering can be applied to the echo signals to obtain heart rate and respiratory rate. Additionally, the processed signal contains motion-related signals, and the energy of motion-related signals can be obtained by performing a fast Fourier transform (FFT) and then multiplying the maximum modulus by the frequency range of interest. When the human body is in motion, the energy of the motion-related signal is usually greater than that when the body is stationary. Therefore, if appropriate energy thresholds are set, Nmr-VSM can recognize the user’s motion status. We set three energy thresholds to recognize four different motion statuses: stillness, relative stillness, motion, and continuous motion. Finally, the distance *R* from the human body to the UWB radar can be calculated using a simple formula $R=\frac{1}{2}ct_{D}$, where *c* represents the speed of light and $t_{D}$ represents the delay in receiving and transmitting electromagnetic waves.

**Data transmission.** All vital sign data obtained after preprocessing the echo signal are uploaded to ECS via WiFi for subsequent correction and application of these data. ECS is a basic computing component composed of CPU (central processing unit), memory, operating system, and cloud disk.

### 3.4. Data Correction

After preprocessing the echo signals, vital sign data of the subject can be obtained. However, at this point, the detected heart rate and respiratory rate are influenced by many factors, leaving a lot of room for improvement in detection accuracy. Therefore, taking the radar heart rate monitoring as an example, we collected real experimental data and designed a DNN-based data correction model to improve the accuracy of vital sign detection.

**Figure 2:**
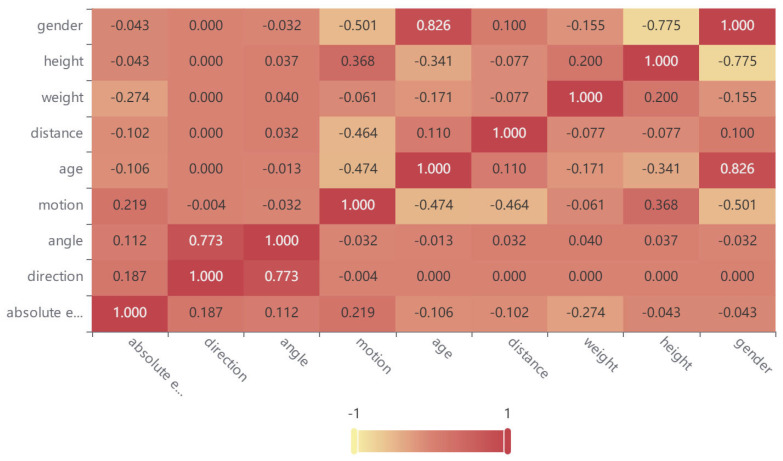
The correlation coefficient heatmap between various factors and the absolute error.

**Figure 3:**
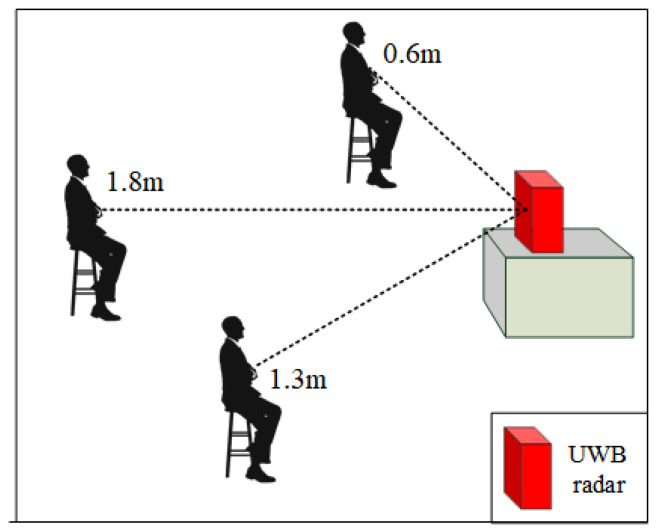
The impact of distance on heart rate detection.

*(1) Factors affecting the accuracy of heart rate monitoring:* We collected real experimental data and analyzed several factors that could have a significant impact on heart rate monitoring. The specific experimental settings will be detailed in Section 4, but in summary, we first recorded the subject’s heart rate simultaneously using a fingertip oximeter and UWB radar under different experimental conditions. We used the oximeter heart rate as the ground truth and calculated the absolute error between the radar heart rate and the oximeter heart rate. Finally, we used Kendall’s tau-b correlation analysis method [25] to analyze the correlation coefficients between various factors and the absolute error. The higher the absolute value of the correlation coefficient, the more significant the impact of the factor on heart rate monitoring is considered. The correlation coefficient heatmap shown in Figure 2 indicates that we analyzed the following factors:

**Distance.** In this paper, distance refers to the straight line distance between the subject and the radar, as shown in Figure 3. However, the distance we tested is far more than what is shown in Figure 3. Since the UWB radar we designed can itself monitor the distance, the subjects can change the distance arbitrarily during the experiment as long as they are within the monitoring range of the radar.

According to Figure 2, the correlation coefficient between distance and absolute error is −0.102, which means that as the distance increases, the absolute error will decrease, and the detection accuracy will improve. However, this conclusion only holds true under specific circumstances, as electromagnetic waves attenuate with distance. When the distance is far enough, the echo signal received by the radar will be severely distorted, affecting the monitoring accuracy. In other words, there exists an optimal monitoring distance range for UWB radar when performing heart rate monitoring, as shown in closed interval (3)

(3) $$\left[L_{\min},L_{\max}\right]$$

where, $L_{\min}$ represents the minimum optimal monitoring distance, and $L_{\max}$ represents the maximum optimal monitoring distance.

When the real monitoring distance *S* is less than $L_{\min}$, increasing *S* can reduce the absolute error and improve the monitoring accuracy. When the real monitoring distance *S* is greater than $L_{\max}$, increasing *S* will increase the absolute error and reduce the monitoring accuracy. When the real monitoring distance *S* is between $L_{\min}$ and $L_{\max}$, changing *S* may either improve or reduce the monitoring accuracy. The values of $L_{\min}$ and $L_{\max}$ are uncertain and are affected by factors such as the environment, the subject’s physical indicators, and hardware equipment.

**Figure 4:**
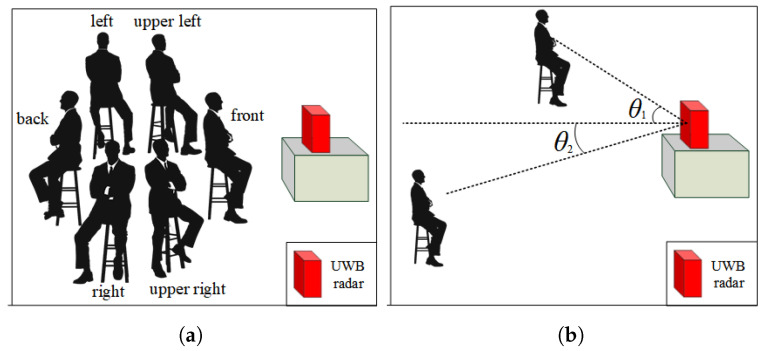
The impact of direction and angle on heart rate detection. (**a**) direction; (**b**) angle.

**Direction and angle.** In this paper, direction refers to the orientation of the subject’s chest relative to the radar, while angle refers to the angle between the line connecting the radar and the subject and the normal vector of the radar array, as shown in Figure 4. The directions tested in this study include front, upper-right, upper-left, right-side, left-side, and back, while the angles tested include 0°, 35°, 45°, 90°, and 180°.

As shown in Figure 2, the correlation coefficients between the absolute error and the angle and direction are 0.112 and 0.187, respectively. This means that as the angle increases, the absolute error will also increase, leading to a decrease in detection accuracy. The same trend is observed with direction: as the subject’s orientation changes from front to back, the detection accuracy decreases. This is due to the fact that the radar’s ability to detect chest rise and fall varies with different directions and angles. When the subject’s chest is directly facing the radar array, chest rise and fall is most easily detected. It is worth noting that, in terms of the correlation coefficients, the detection accuracy is higher when the direction is to the right than when it is to the left. This is due to physiological differences in the human body: the chest rise and fall caused by heartbeats is more pronounced on the left side of the chest.

**Subject physical characteristics.** In this study, subject physical characteristics include gender, weight, height, and age. As shown in Figure 2, the correlation coefficients between these four physical characteristics and the absolute error are all negative. The smallest correlation coefficient is between weight and absolute error, which is −0.274. Gender and height have the largest correlation coefficients with the absolute error, both at −0.043. Age has a moderate correlation with the absolute error, with a coefficient of −0.106. Weight has a greater impact on detection accuracy because as weight increases, chest fat thickness also increases, leading to the masking of chest rise and fall caused by heartbeats.

**Motion status.** The principle of detecting heart rate using UWB radar is to detect chest rise and fall caused by heartbeats, which are typically very weak, usually only 4–12 mm in amplitude. On the other hand, body motions caused intentionally or unintentionally by the subject are usually at a centimeter level or above, much greater than the amplitude of chest rise and fall, and therefore, subject’s body motion can potentially have a significant impact on heart rate detection, even completely masking the heartbeat signal, leading to abnormal results. In this study, experiments were conducted under four motion statuses detected by UWB radar, including stillness, relative stillness, motion, and continuous motion.

As shown in Figure 2, the correlation coefficient between motion status and absolute error is 0.219. As expected, motion status has a significant impact on the absolute error, with greater subject body motion resulting in larger absolute error and lower detection accuracy.

**Figure 5:**
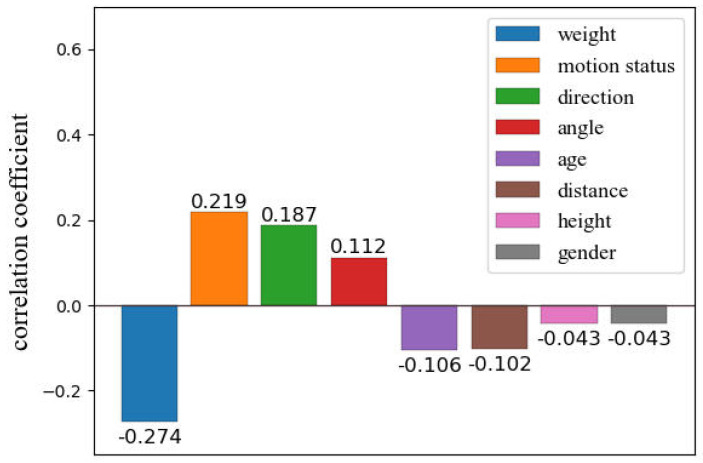
Comprehensive analysis of the impact of various factors on heart rate detection.

**Comprehensive analysis.** We evaluated the impact of the above eight factors on heart rate detection and found that weight has the most significant impact on detection accuracy, followed by motion status, while gender and height have the weakest impact. From the bar chart shown in Figure 5, the impact of these factors on detection accuracy decreases sequentially from left to right.

Some factors have a very weak impact on detection accuracy, such as gender and height, with correlation coefficients of −0.043. However, considering the uncertainty of the parameters $L_{\max}$ and $L_{\min}$ in the optimal detection distance range $\left[L_{\min},L_{\max}\right]$ of UWB radar, changes in gender and height may affect the parameters, so it is necessary to include gender and height as inputs to the data correction model.

*(2) Data correction based on DNN:* To eliminate the impact of the above eight factors on heart rate detection, we designed a data correction model based on DNN. The input of this model includes oximeter heart rate, radar heart rate, direction, angle, gender, height, weight, age, distance and motion status, and the output is the corrected radar heart rate. The training dataset for the model is derived from the data collected in our experiments, and we aim to make the corrected radar heart rate as close as possible to the oximeter heart rate.

**Figure 6:**
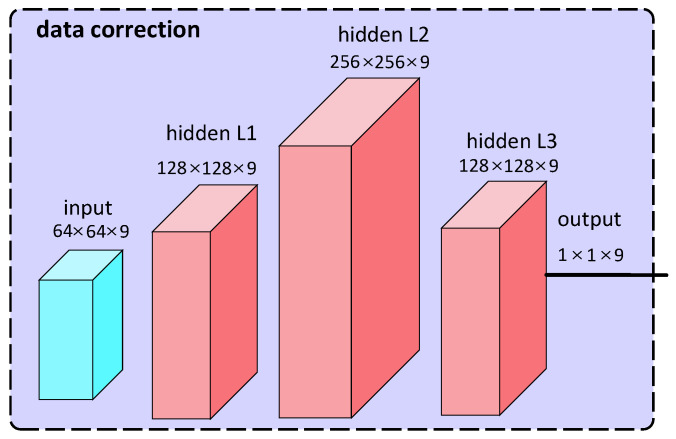
The architecture of the data correction model.

As shown in Figure 6, our designed DNN consists of an input layer, three hidden layers, and an output layer. Specifically, the input layer is responsible for receiving the input feature data and passing it to the next layer. The input layer’s input dimension is 9, including 64 neurons. Then, the feature data processed by the input layer are directly transmitted to the first hidden layer, and then passed to the second hidden layer, and so on. The three hidden layers of the DNN all use PReLU activation functions to enhance the model’s nonlinear expression ability, making it easier for the model to capture complex data patterns. Meanwhile, to avoid overfitting, we also apply the Dropout strategy to the three hidden layers of the model, randomly deactivating a certain proportion of neurons during model training. The differences among these three hidden layers lie in their neuron numbers and dropout rates. The first hidden layer contains 128 neurons with a dropout rate of 0.2; the second hidden layer contains 256 neurons with a dropout rate of 0.3; while the third hidden layer contains 128 neurons with a dropout rate of 0.4. Finally, there is the output layer of the DNN, which contains one neuron responsible for predicting the heart rate correction value.

To measure the gap between the predicted values of the model and the true values (i.e., the oximeter heart rate), we use the MSE (mean squared error) as the loss function:

(4) $$MSE=\frac{1}{n}\sum_{i=1}^{n}{\left(y_{i}-{\hat{y}}_{i}\right)}^{2}$$

where, ${\hat{y}}_{i}$ represents the predicted value, $y_{i}$ represents the true value, and *n* represents the number of samples.

To more effectively handle sparse matrices, accelerate the training process, and improve the model’s robustness, we employed the Adagrad optimizer. This is an adaptive learning rate optimization method that dynamically adjusts the learning rate based on the gradient history of each parameter during training. Its update formula is as follows:

(5) $$\begin{array}{c} g_{t,i}=\nabla_{\theta }J\left(\theta_{i}\right)\\ G_{t,ii}=G_{t-1,ii}+g_{t,i}^{2}\\ \theta_{t+1,i}=\theta_{t,i}-\frac{\eta }{\sqrt{G_{t,ii}+\varepsilon }}g_{t,i} \end{array}$$

where, $g_{t,i}$ represents the gradient of $\theta_{i}$ at time *t*. We use $G_{t,ii}$ to denote an element in the *i*-th row and *i*-th column of the diagonal matrix $G_{t}$, which is the sum of squares on the historical gradients of $\theta_{i}$ at time *t*. ε is a very small value to avoid the denominator being 0. η is a hyperparameter, representing the global learning rate.

### 3.5. Anomaly Detection

**Figure 7:**
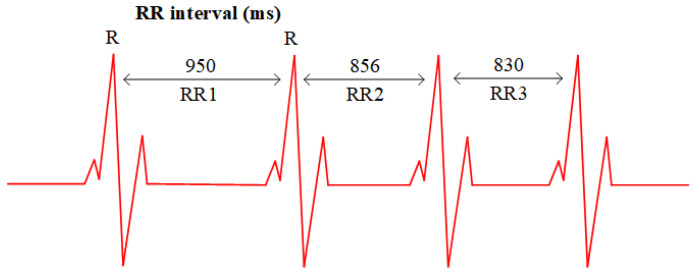
RR1, RR2, and RR3 represent the intervals between successive R-peaks on the ECG signal, while HRV refers to the time variation between these adjacent RR intervals.

After being corrected by the DNN model described earlier, we obtain a heart rate value that is closer to the true heart rate. However, simply presenting the heart rate in numerical form does not have a significant impact on users’ health monitoring, especially for non-medical people who have difficulty in extracting useful information from a large amount of heart rate data. To address this issue, we leveraged the corrected heart rate data to design a CNN-based HRV analysis model that enables anomaly detection for users. The details are explained below:

**HRV.** The time variation between successive heartbeats is defined as HRV, which arises from modulation of the sinoatrial node by the autonomic nervous system (ANS), leading to fluctuations or differences in the order of tens of milliseconds in the heartbeat intervals [26]. From an electrocardiogram perspective, HRV refers to the temporal changes that occur within a continuous sequence of RR intervals, where the RR interval is the time between two consecutive R waves, as shown in Figure 7. HRV may serve as an indicator of current diseases or as an early warning sign of impending cardiac conditions [27]. Additionally, HRV can be applied in emotional analysis [28], sleep staging [29], and other areas.

**Dataset.** We trained our CNN model on a publicly available dataset [30,31,32]. Specifically, the dataset [30] comprises 135 sets of RR interval sequences recorded by an implanted cardioverter-defibrillator (Medtronic Jewel PlusTM ICD 7218) from 78 patients with spontaneous ventricular tachyarrhythmia. When the ICD detects ventricular tachycardia or ventricular fibrillation in a patient, it applies programmed pacing, cardioversion, or defibrillation therapy. Additionally, the ICD records 1024 consecutive RR intervals before the occurrence of abnormal events, with the first detection of the abnormal event serving as the endpoint. On the other hand, the datasets [31,32] consist of RR interval sequences detected using ECG technology from healthy individuals, and these RR interval sequences are entirely normal.

**CNN-based anomaly detection.** Our designed model achieves a binary classification task of anomaly detection, with 1024 consecutive RR intervals as input and the output being “abnormal” or “normal”.

**Figure 8:**
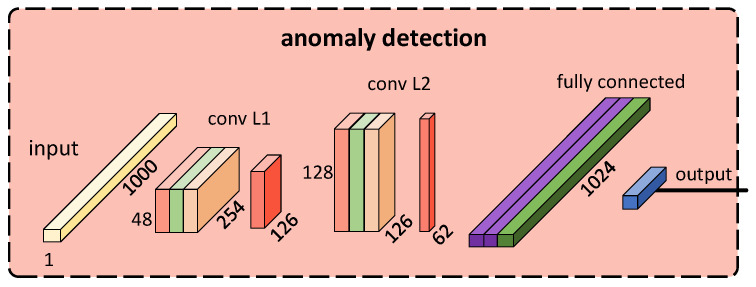
The architecture of the anomaly detection model.

The model consists of three sub-network layers, including two convolutional layers and one fully connected layer. Specifically, each convolutional layer is composed of a Conv1d layer, a BatchNorm1d layer for batch normalization, a ReLU activation function, and an AvgPool1d layer for down-sampling, to extract features from the RR interval sequence. The fully connected layer includes two hidden layers and an output layer, where each hidden layer contains a linear fully connected layer and a ReLU activation function. The first hidden layer also includes a Dropout regularization layer to prevent overfitting. Finally, a linear layer acts as the classification layer in the fully connected layer to output the prediction results. The structure of the model is illustrated in Figure 8.

To measure the predictive performance of the model, we utilized the cross-entropy loss function:

(6) $$L=-\frac{1}{N}\sum_{i=1}^{n}y_{i}\log{\hat{y}}_{i}+\left(1-y_{i}\right)\log\left(1-{\hat{y}}_{i}\right)$$

where, *N* represents the number of samples, used to average the loss function of all samples. $y_{i}$ represents the true label of the *i*-th sample, which is a binary variable, taking values of 0 or 1, representing that the sample belongs to the negative class or the positive class. ${\hat{y}}_{i}$ represents the probability of the *i*-th sample being predicted as the positive class.

Then, to effectively avoid gradient vanishing or explosion, improve model robustness and convergence speed, we employed the Adam optimizer, whose update formula is as follows:

(7) $$\begin{array}{c} m_{t}=\beta_{1}m_{t-1}+\left(1-\beta_{1}\right)g_{t}\\ v_{t}=\beta_{2}v_{t-1}+\left(1-\beta_{2}\right)g_{t}^{2}\\ {\hat{m}}_{t}=\frac{m_{t}}{1-\beta_{1}^{t}}\\ {\hat{v}}_{t}=\frac{v_{t}}{1-\beta_{2}^{t}}\\ \theta_{t+1}=\theta_{t}-\alpha \frac{{\hat{m}}_{t}}{\sqrt{{\hat{v}}_{t}+\varepsilon }} \end{array}$$

where, $g_{t}$ represents the gradient at step *t*. $\beta_{1}$ and $\beta_{2}$ respectively represent the exponential decay rates of the first-order moment and the second-order moment. $m_{t}$ and $v_{t}$ respectively represent the first-order moment estimate and the second-order moment estimate at step *t*. ${\hat{m}}_{t}$ and ${\hat{v}}_{t}$ respectively represent the bias-corrected first-order moment estimate and the second-order moment estimate at step *t*. $\theta_{t}$ represents the model parameters at step *t*. α represents the learning rate. ε is a very small value to avoid dividing by zero.

**Model latency value k.** Due to each group of data in the abnormal dataset [30] used to train the model being a sequence of 1024 consecutive RR intervals, the model must accumulate 1024 or more consecutive RR intervals before it can perform analysis. In other words, if there is an RR interval sequence:

(8) $$X=\left\{x_{1},x_{2},x_{3},\ldots ,x_{n-1},x_{n}\right\}$$

where, *X* represents *n* consecutive RR interval sequences, $x_{n}$ represents the *n*-th RR interval, and *n* is greater than or equal to 1024.

Then, when the model analyzes sequence (8), it will first input a subsequence of sequence (8):

(9) $$X_{sub1}=\left\{x_{1},x_{2},\ldots ,x_{1023},x_{1024}\right\}$$

Subsequently, the model will output the analysis result of $X_{sub1}$, i.e., whether the RR interval $x_{1024}$ is abnormal or not.

And then, the model will input the second subsequence.

(10) $$X_{sub2}=\left\{x_{2},x_{3},\ldots ,x_{1024},x_{1025}\right\}$$

And subsequently, the model will output the analysis result of $X_{sub2}$, i.e., whether the RR interval $x_{1025}$ is abnormal or not.

This process will be repeated for each subsequent subsequence until the final 1024 RR interval sequence is analyzed.

However, considering that the model needs to comprehensively analyze the 1024 RR interval sequences, and in some abnormal cases, the changes in the RR intervals may not be obvious, the model’s detection of anomaly, therefore unavoidably has a certain latency. From this point on, we can define the latency value *k* as follows:

**Definition** **1** **(latency value** k**).*** Assuming there is an abnormal segment $\left\{x_{i},x_{i+1},\ldots ,x_{j-1},x_{j}\right\}$ in the RR interval sequence (8), as shown below:*

(11) $$X=\left\{x_{1},x_{2},\ldots ,x_{i},x_{i+1},\ldots ,x_{j-1},x_{j},\ldots ,x_{n}\right\}$$

*where, j>i≥1024.*

*The model should detect this abnormal segment for the first time when it inputs the sequence (12) and ouputs the analysis result of it.*

(12) $$X_{sub(i+k-1023)}=\left\{x_{i+k-1023},\ldots ,x_{i+k-1},x_{i+k}\right\}$$

*where, k is a positive integer known as the latency value k.*

In other words, the anomaly detection model can only identify a specific abnormal segment after a delay of *k* RR intervals, such as abnormal segment in sequence (11). The delay value *k* is represented here by *k*.

### 3.6. Demonstration

**Figure 9:**
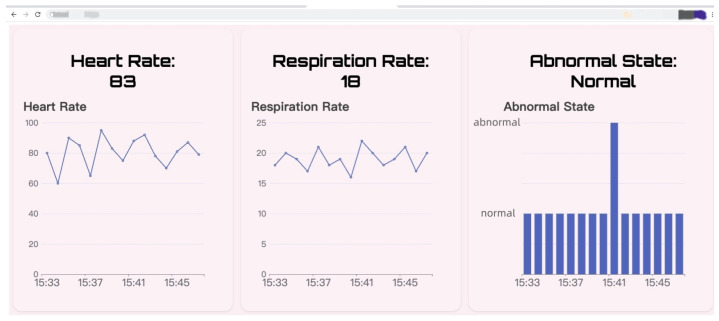
Demonstration of the Nmr-VSM.

To visualize the results of vital sign monitoring and anomaly detection, and to enable users to easily understand the subject’s physical condition, we designed a web page with front-end and back-end separation. The back-end of this web-page is implemented based on the Spring Boot framework while the front-end is based on mainstream frameworks such as jQuery.js and Echarts.js. Additionally, bidirectional communication between the front-end and back-end is achieved through WebSocket. This webpage is deployed on ECS, and users can access it via a web browser to view the subject’s vital signs in real-time. As shown in Figure 9, users can visually observe the subject’s heart rate and respiration rate on this web-page.

## 4. Evaluation

In this section, we evaluate the data correction model and the anomaly detection model.

### 4.1. Data Correction Model

**Figure 10:**
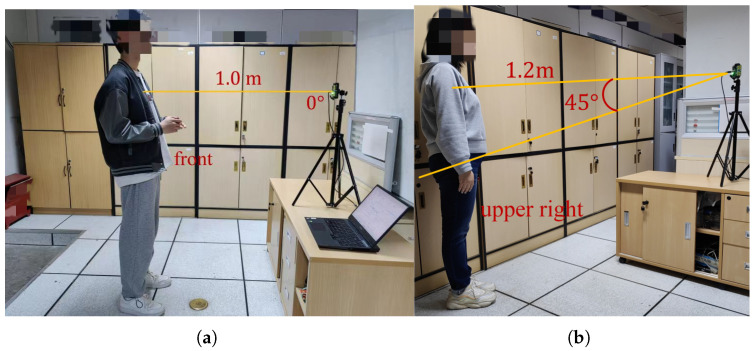
Experimental setup. (**a**) subject A is undergoing the experiment; (**b**) subject B is undergoing the experiment.

**Experimental setup.** We collected a total of 10,400 data points through real experiments to train and evaluate the model. The data were obtained from seven subjects, where each data point included the subject’s direction, angle, distance relative to the UWB radar, gender, height, weight, age, motion status, heart rate measured by the fingertip oximeter, and heart rate measured by the UWB radar. During the experiment, we randomly varied the subject’s direction, angle, distance, and motion status while monitoring their heart rate using the UWB radar. Additionally, the subjects wore a fingertip oximeter that provided the true heart rate value, as shown in Figure 10. All the above-mentioned metrics were used as inputs for the data correction model. Finally, the model outputted the corrected heart rate value.

**Evaluation method.** We referred to the heart rate detected by the fingertip oximeter as the true value, the heart rate detected by the UWB radar as the measured value, and the heart rate outputted by the model as the corrected value. We used the following metrics to evaluate the performance of the model:

The first metric is the mean absolute error (MAE):

(13) $$MAE=\frac{1}{n}\sum_{i=1}^{n}\left|y_{i}-{\hat{y}}_{i}\right|$$

where $y_{i}$ represents the true value, ${\hat{y}}_{i}$ represents the predicted value, and *n* represents the sample size. A smaller MAE indicates better performance of the model.

The second metric is the mean relative error (MRE):

(14) $$MRE=\frac{1}{n}\sum_{i=1}^{n}\frac{\left|y_{i}-{\hat{y}}_{i}\right|}{\left|y_{i}\right|}$$

Similarly, $y_{i}$ represents the true value, ${\hat{y}}_{i}$ represents the predicted value, and *n* represents the sample size. The MRE has a range between 0 and 1, exclusive, with a smaller value indicating better performance of the model.

The third metric is the mean squared error (MSE), which is shown in Equation (4).

**Figure 11:**
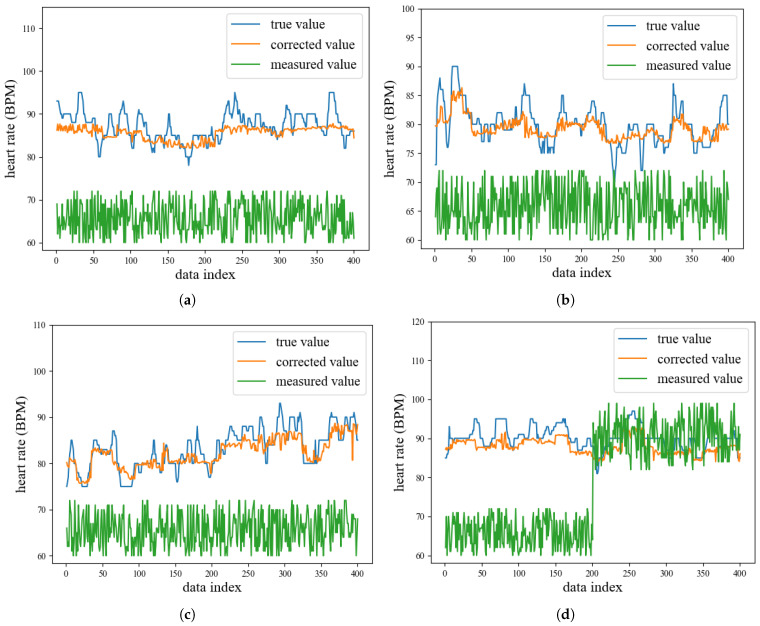
Evaluation results of the data correction model. (**a**–**d**) represent the true value, corrected value, and measured value for subjects 1–4, respectively.

**Figure 12:**
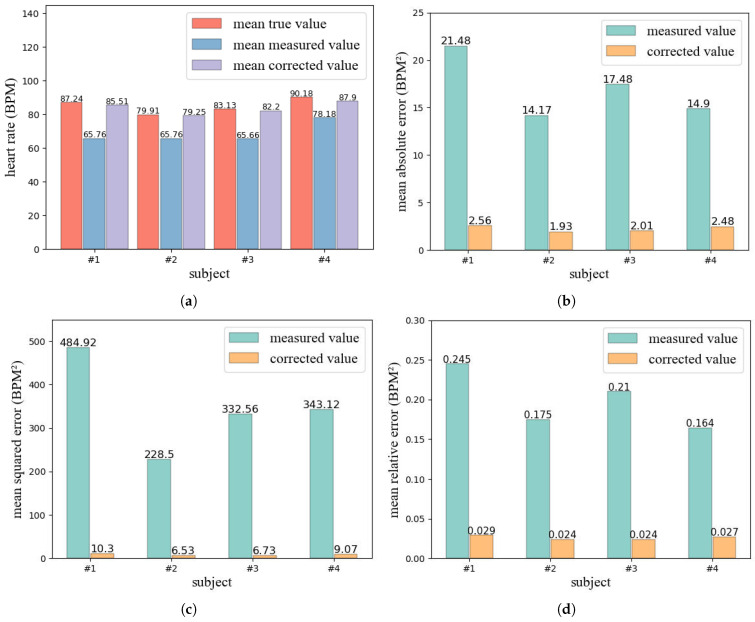
Evaluation results of the data correction model. (**a**) represents the mean true value, mean measured value, and mean corrected value for subject 1. (**b**) shows the MAE of the measured value and corrected value for subject 1. (**c**) shows the MSE of the measured value and corrected value for subject 1. (**d**) shows the MRE of the measured value and corrected value for subject 1.

**Evaluation results.** We randomly selected four subjects out of the seven, and for each subject, 400 data points were collected for model evaluation. As shown in Figure 11a–d and Figure 12a, the measured values differ significantly from the true value in most cases, and exhibit random fluctuations with little relation to the true value. In contrast, the corrected values tend to approximate the true value well, with a trend that closely follows the true value. Additionally, as shown in Figure 12b–d, the error of the corrected value is much lower than that of the measured value. For example, for subject 1, the MAE, MSE and MRE of the measured value reached 21.48, 484.92, and 0.245 respectively. In comparison, the MAE of the corrected value was only 2.56, representing an 88.08% reduction. Furthermore, the MSE and MRE of the corrected value were only 10.30 and 0.029, respectively, indicating a 97.88% and 89.39% decrease compared to the measured value.

Overall, our designed data correction model achieved good performance and significantly reduced the errors. The model can accurately correct the measured value to near the true value, with a trend that closely matches the true value.

### 4.2. Anomaly Detection Model

**Experimental setup.** Since all seven subjects in this study were completely healthy and had no heart disease, we randomly selected one subject from the seven and recorded 1600 consecutive RR intervals for this subject, which were all considered normal. Then, we inserted six abnormal segments randomly into the 1024th to 1600th RR intervals to simulate heart diseases such as ventricular tachycardia or ventricular fibrillation. These abnormal segments varied in severity, with some having more pronounced fluctuations than others. Finally, we used the model to detect the abnormal segments and evaluated the detection performance.

**Evaluation method.** We evaluate the model by setting a reasonable maximum latency value k. Specifically, since for most people, their heart rate is usually higher than 60 BPM, when the latency value *k* is equal to 10, the maximum latency time for detecting an abnormal segment will not exceed 10 s. This is entirely acceptable.Therefore, we consider a successful detection when the latency value *k* for detecting an abnormal segment is less than or equal to 10.

**Evaluation results.** The evaluation results are shown in Table 2. All abnormal segments inserted were successfully detected by the model, and the latency values *k* for these six segments were relatively small. The smallest *k* was only 1, which occurred in an abnormal segment with an average RR interval of 299.5 ms. The largest *k* was 8, which occurred in an abnormal segment with an average RR interval of 502.

Overall, the anomaly detection model we designed can achieve good performance and successfully detected abnormal situations at a lower latency.

## 5. Conclusions

In this paper, we propose a novel non-touch vital sign monitoring system Nmr-VSM based on UWB radar by considering the influence of multiple variables and modeling HRV of the human body. Firstly, we design a UWB radar that can perform multi-dimensional vital sign monitoring, including heart rate, respiratory rate, distance, and motion status. Based on this, we propose a DNN-based heart rate data correction model by analyzing real experimental data. Then, we model HRV of the human body and design a CNN-based cardiac anomaly detection model. Finally, we evaluate the performance of Nmr-VSM through extensive experiments. The results demonstrate that Nmr-VSM can effectively reduce the error in heart rate monitoring and achieve low-latency detection of cardiac anomalies.

In future work, we will consider correcting the respiration rate measured by radar. Additionally, we will further consider incorporating more types of data to train the anomaly detection model, enabling detection of a wider range of heart diseases.Finally, we will also consider implementing real-time vital sign monitoring for multiple individuals.

## Figures and Tables

**Table 1:** Frequency and amplitude of chest rise and fall caused by respiration and heartbeat.

Category	Frequency (HZ)	Amplitude (mm)
respiration	0.1–0.3	4–12
heartbeat	1–2	0.2–0.5

**Table 2:** Evaluation results of the anomaly detection model.

Abnormal Segment	Average RR Interval (ms)	Latency Value *k*	Detection Result
1	350	7	success
2	315	6	success
3	450	7	success
4	350	5	success
5	299.5	1	success
6	502	8	success

## Data Availability

Due to privacy and ethical restrictions, we are unable to provide access to the data used in this study. This decision was made to protect the confidentiality and privacy of the study participants. We understand that access to data is important for scientific progress, but we believe that protecting the privacy of our participants is paramount. We apologize for any inconvenience this may cause and hope that our findings will still be valuable to the scientific community.
